# The dark side of foetal bovine serum in extracellular vesicle studies

**DOI:** 10.1002/jev2.12271

**Published:** 2022-10-10

**Authors:** Ornella Urzì, Roger Olofsson Bagge, Rossella Crescitelli

**Affiliations:** ^1^ Sahlgrenska Center for Cancer Research and Wallenberg Centre for Molecular and Translational Medicine Department of Surgery Institute of Clinical Sciences Sahlgrenska Academy University of Gothenburg Gothenburg Sweden; ^2^ Department of Biomedicine Neurosciences and Advanced Diagnostics (Bi.N.D) University of Palermo Palermo Italy; ^3^ Department of Surgery Sahlgrenska University Hospital Region Västra Götaland Gothenburg Sweden

**Keywords:** cell culture, contaminants, extracellular vesicles, foetal bovine serum

## Abstract

Extracellular vesicles (EVs) have been shown to be involved in cell‐cell communication and to take part in both physiological and pathological processes. Thanks to their exclusive cargo, which includes proteins, lipids, and nucleic acids from the originating cells, they are gaining interest as potential biomarkers of disease. In recent years, their appealing features have been fascinating researchers from all over the world, thus increasing the number of in vitro studies focused on EV release, content, and biological activities. Cultured cell lines are the most‐used source of EVs; however, the EVs released in cell cultures are influenced by the cell culture conditions, such as the use of foetal bovine serum (FBS). FBS is the most common supplement for cell culture media, but it is also a source of contaminants, such as exogenous bovine EVs, RNA, and protein aggregates, that can contaminate the cell‐derived EVs and influence their cargo composition. The presence of FBS contaminants in cell‐derived EV samples is a well‐known issue that limits the clinical applications of EVs, thus increasing the need for standardization. In this review, we will discuss the pros and cons of using FBS in cell cultures as a source of EVs, as well as the protocols used to remove contaminants from FBS.

## 1 INTRODUCTION

Extracellular vesicles (EVs) are membrane‐bound nanoparticles naturally released in the extracellular space by almost all cell types (Van Niel et al., 2018). Although initially considered a mechanism by which the cell excretes waste products (Harding et al., 1983; Pan & Johnstone, 1983), in recent years EVs have been shown to play a key role in cell‐cell communication (Colombo et al., 2014; Lo Cicero et al., 2015; Yáñez‐Mó et al., 2015). EVs can carry numerous active biomolecules, such as proteins (Doyle & Wang, 2019), nucleic acids (Ridder et al., 2014) (including DNA, mRNAs, miRNAs, (Valadi et al., 2007) and lncRNAs (Conigliaro et al., 2015)) and lipids (Dang et al., 2017), that can be transferred to target cells and modulate their phenotypes and functional properties (Valadi et al., 2007). EVs have been classified based on their size and biogenesis into three main categories: (i) exosomes, (ii) microvesicles, and (iii) apoptotic bodies (Crescitelli et al., 2013; EL Andaloussi et al., 2013). Exosomes are the smallest EVs (∼40–120 nm in diameter) and they originate from the endosomal compartment following the fusion of multivesicular bodies with the cell membrane (French et al., 2017). Microvesicles are usually from 100 nm to 1 μm in diameter and are formed directly by the outward budding of the plasma membrane (Cocucci et al., 2009). Finally, apoptotic bodies are the largest EVs, ranging from ∼500 nm to 5 μm, and are released as a consequence of programmed cell death (Xu et al., 2019). However, as demonstrated in several studies (Kowal et al., 2016; Lässer et al., 2017; Lázaro‐Ibáñez et al., 2019; Osteikoetxea et al., 2015) EV subpopulations cannot be classified exclusively based on size because there is a dimension overlap among EV subpopulations. Results from several studies focusing on analysing RNA and protein content have demonstrated that EV subpopulations show stronger similarity based on their density than on size, both at the protein and RNA levels (Crescitelli et al., 2020; Lässer et al., 2017). Thus the best way to classify EV subpopulations is still an open discussion in the EV community due to the lack of standardized protocols to separate them.

EVs have a role in both physiological and pathological processes (Raposo & Stoorvogel, 2013; Yáñez‐Mó et al., 2015). One of the first functions attributed to EVs was antigen presentation, as demonstrated by Raposo and colleagues (Raposo et al., 1996). The involvement of EVs in immune response has been heavily studied, and nowadays it is known that immune cell‐derived EVs mediate the exchange of co‐stimulatory molecules (Bobrie et al., 2011), miRNAs (Mittelbrunn et al., 2011; Montecalvo et al., 2012), cytokines (Qu et al., 2007), and other biomolecules involved in inflammatory response, differentiation, and immunosuppression (Awadasseid et al., 2021; Quaglia et al., 2020; Robbins & Morelli, 2014). On the other hand, EVs also take part in the development of neurodegenerative diseases such as Alzheimer's disease (Miranda et al., 2018) and Parkinson's disease (Danzer et al., 2012), inflammatory diseases (Mossberg et al., 2017; Surmiak et al., 2021), and ageing (Schwartz et al., 2021; Tanaka & Takahashi, 2021). An increasing number of studies have shown the involvement of EVs released by many kinds of cells present in the tumour microenvironment, including both cancer and normal cells, in every step of tumour progression, including tumour initiation (Afify et al., 2022), growth (Gan et al., 2013), and metastasis (Keerthikumar et al., 2015). Moreover, EVs can be isolated from numerous biological fluids such as blood (Caby et al., 2005; Xu et al., 2021), saliva (Aqrawi et al., 2017; Comfort et al., 2021), cerebrospinal fluid (Muraoka et al., 2020; Norman et al., 2021), malignant effusions (Andre et al., 2002), seminal fluid (Roca et al., 2021), urine (Merchant et al., 2017; Pisitkun et al., 2004), and breast milk (Admyre et al., 2007). The EV content reflects the contents of the originating cell so that they can be used as biomarkers for diseases (De Freitas et al., 2021; Simeone et al., 2020; Urabe et al., 2020), and increasing evidence has shown how EVs can be useful tools in the context of liquid biopsies (Ciferri et al., 2021; Zhou et al., 2020). EVs have been investigated as biomarkers in cancer because they are shed into the circulation from primary tumors (Melo et al., 2015; Zhang et al., 2017), because they can potentially be used for the early detection of metastasis (Guan et al., 2015; Saugstad et al., 2017), and because they can provide information about the disease through a easily obtained biological fluid like blood, urine, or saliva (Poulet et al., 2019). The cargo of EVs can also be used as biomarkers for neurodegenerative diseases (Hornung et al., 2020; Saugstad et al., 2017), as was demonstrated for Alzheimer's (Lee et al., 2019) and Parkinson's diseases (Russo et al., 2012), and for cardiovascular diseases (Huang et al., 2021; Lenart‐Migdalska et al., 2021).

Many research groups have been investigating the possibility of using EVs as nanocarriers for drug delivery (Herrmann et al., 2021) given their similarity to liposomes. Another promising feature of EVs as a drug delivery system is their safety (Escudier et al., 2005) due to their lack of toxicity or immunogenicity (Zhu et al., 2017), and their complex composition might ensure tissue or organ homing (EL Andaloussi et al., 2013). Moreover, they can cross biological barriers, such as the blood brain barrier (Alvarez‐Erviti et al., 2011; El‐Andaloussi et al., 2012), and their stability allows for the successful delivery of drugs both locally and at distant sites (Herrmann et al., 2021). Zhang et al. demostrated that EVs isolated from red blood cells (RBC‐EVs) naturally accumulated in the liver of mice following intravenous injection, showing that EVs represent a possible drug vehicle for liver diseases (Zhang et al., 2020). RBC‐EVs loaded with an antisense oligonucleotide of microRNA‐155, which was reported to alleviate liver injury (Yang et al., 2018), showed protective effects against acute liver failure in vivo by inhibiting microRNA‐155 expression and promoting M2 macrophages polarization in the liver (Zhang et al., 2020). In addition, Tian et al. developed a system for the targeted delivery of doxorubicin, a chemotherapeutic drug, to breast cancer cells using mouse immature dendritic cell‐derived EVs. They engineered mouse immature dendritic cells to express Lamp2, a known exosomal membrane protein, fused with an iRGD‐targeting peptide, and after EV isolation they loaded EVs with doxorubicin (iRGD‐Exos‐Dox). The iRGD‐Exos‐Dox showed higher antitumor activity against breast cancer compared to the free drug both in vitro and in vivo and with limited side effects (Tian et al., 2014). The study of EVs in vitro will increase our knowledge of physio‐pathological mechanisms and will provide new therapeutic platforms.

**FIGURE 1:**
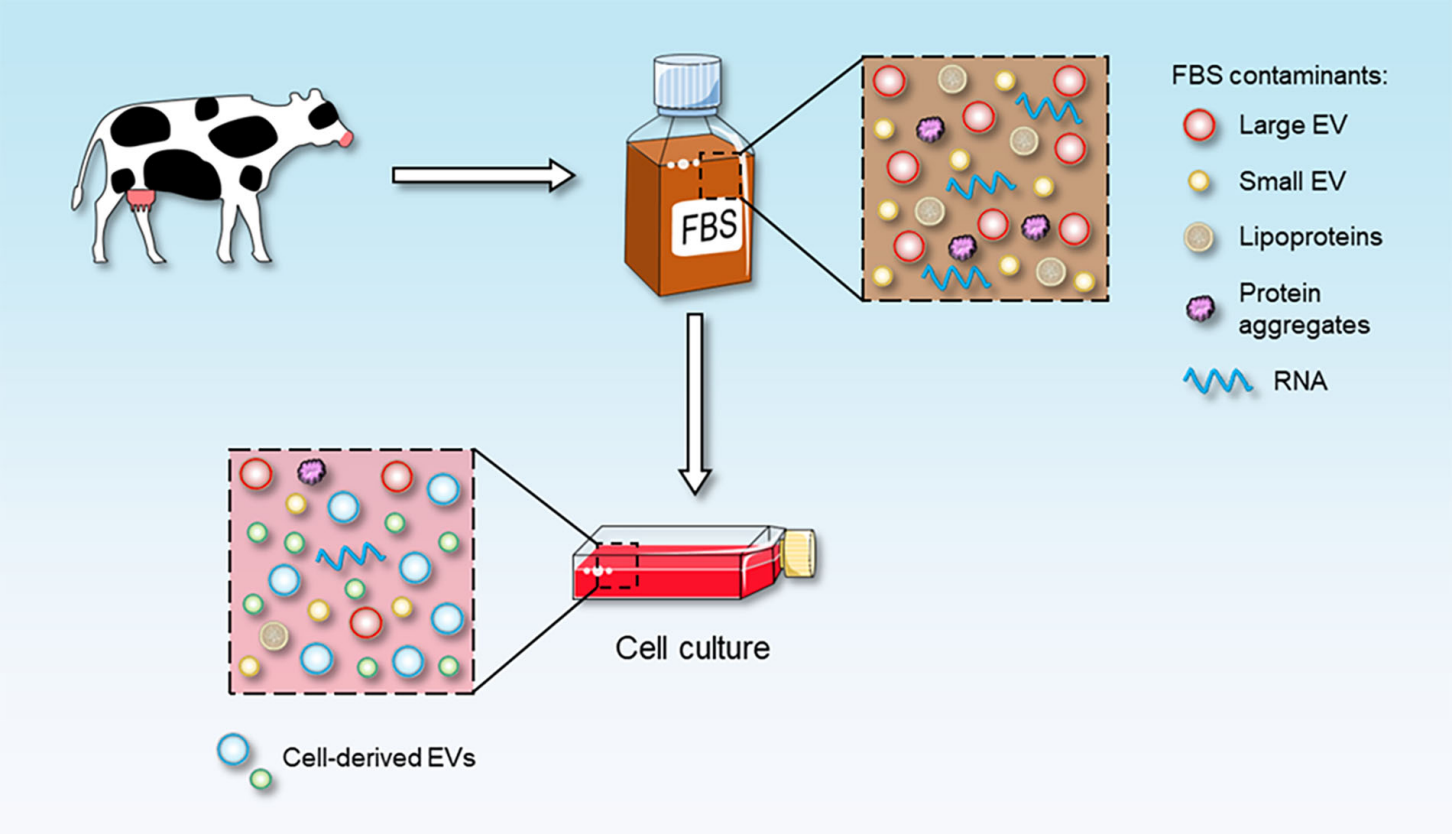
FBS‐derived contaminants in cell‐derived EV samples

Nevertheless, the standardization of methods used to separate EV subpopulations still represents a great challenge that needs to be solved. The appropriate distinction of the various EV subpopulations could prevent cross‐contamination between different EV categories, thus avoiding the misinterpretation of data regarding EV biogenesis, content, and functions. Differential ultracentrifugation still represents the gold standard for EV isolation; however, with this technique the EV pellet may be contaminated by other particles, such as lipoprotein particles and protein aggregates, which coprecipitate with EVs (Witwer et al., 2013). On the contrary, the density gradient separation is able to remove lipoprotein particles that are similar in size of EVs but different in density (Yuana et al., 2014) and the size exclusion chromatography separate EVs from soluble proteins (Karimi et al., 2018). Although much work remains to be done, in the last years new techniques have been developed to overcome these contamination problems; the combination of different methods has proven to be the best solution to isolate EV subpopulations with high purity (Gandham et al., 2020; Karimi et al., 2018). Another important limitation to the use of EVs for diagnosis and therapy is the lack of standardization of the cell culture conditions for EV isolation. For instance, authors should report the cell viability at the time of conditioned media collection for EV isolation because dead cells can release membrane structures that would overestimate the true number of EVs (Théry et al., 2018), and they should report other information like cell passage, seeding density, and cell confluence at the time of collection of conditioned media (Patel et al., 2017). Moreover, another crucial parameter is the culture media composition because the use of specific supplements (Zhou et al., 2017) can influence EV release and EV contents (Théry et al., 2018). The main supplement used in cell culture growth media is foetal bovine serum (FBS), which might limit the clinical applications of cell line‐derived EVs because FBS contamination in the EV preparation might induce xeno‐immunization or involuntary transmission of zoonotic agents (Dessels et al., 2016; Kang et al., 2020). It is well known that FBS itself contains EVs (Figure 1), and therefore it is recommended to deplete EVs from FBS before adding it to cell culture media (Théry et al., 2006).

In this review, we will discuss how the use of FBS can affect cell behaviour and thus can affect EV release and purity. We will also summarize the methods to obtain pure and clear EV samples from cell culture supernatant with the aim of avoiding FBS‐derived EV contaminations.

## 2 FBS: PROS AND CONS FOR CELL CULTURES

The term “cell culture” indicates the methods by which cells are grown outside a living organism under controlled conditions (e.g., pH, temperature, and osmolarity) (Philippeos et al., 2012). The cell culture technique represents a fundamental tool in many fields such as biological research, biotechnology, and assisted reproductive technology (Leist et al., 2008; Park & Eve, 2009). The cells are maintained in culture by using culture media and supplements that favour cell growth. In 1958, Puck et al. introduced the use of FBS in cell cultures to stimulate cellular growth (Puck et al., 1958). Nowadays, FBS is one of the most important supplements of the basal media of primary cells, as well as cell lines from both human and other mammalian species. FBS contains amino acids, proteins, vitamins, growth factors, inorganic salts, carbohydrates, and lipids, all of which support cell growth and are essential for cell attachment and maintenance (Gstraunthaler, 2003) thus making FBS a universal supplement for cell culture media.

The concentration of FBS in cell growth media varies from 2% to 20% depending on cell type, although the most commonly used is 10%, and this parameter is crucial because it can affect cell proliferation. For instance, it was demonstrated that the proliferation of human bone marrow‐derived mesenchymal stem cells (BM‐MSCs) was directly proportional to the percentage of FBS in culture media. The BM‐MSC proliferation increased in a concentration‐dependent manner when the concentration of FBS increased from 5% to 20% (Khasawneh et al., 2019). On the other hand, MSC production of immunosuppressive molecules, such as interleukin 10 and indoleamine 2,3‐dioxygenase 1, were optimal with 7% and 10% of FBS, while with 5% or 20% FBS the production was reduced, thus emphasizing the importance of choosing the correct concentration of FBS for MSC maintenance in culture according to the purpose of the experiments to be conducted (Shanbhag et al., 2017). Interestingly, FBS can also enhance the efficiency of cell transduction, as was demonstrated by Abbasalipour et al. (Abbasalipour et al., 2019). They found that the coating of plates with FBS before cell seeding increased the number of K562 cells (human lymphoblasts) attached and that the lentiviral transduction efficiency increased compared to uncoated conditions (Abbasalipour et al., 2019). FBS is also widely used as a chemoattractant in cell migration assays; nevertheless, its presence in cell culture media can influence the results of these tests (Akasaka et al., 2005; Lin et al., 2014). Usually, to perform a migration assay the lower chamber should be full of media supplemented with 2% to 10% FBS, while the transwell insert is filled with serum‐free media to form a gradient (Li et al., 2011; Zhen et al., 2017; Zhu et al., 2016). It has been demonstrated that the migration ability of BV2 microglial cells is promoted by the presence of 5% FBS, even in the absence of a serum gradient, thus potentially confounding the results of the transwell migration assay (Omar Zaki et al., 2019).

The use of FBS in cell culture carries disadvantages related to the presence of contaminants, such as endotoxins, mycoplasma, viruses, and prion proteins, but also due to the undefined composition that varies from lot to lot (Baker, 2016; Bryan et al., 2011). As a demonstration of this, three lots of FBS from different manufacturers were shown to differentially promote the growth of adult cultured pigmented retinal epithelial cells (Zheng et al., 2006). Through proteomic analysis, it was found that the lot of FBS with the highest growth‐promoting ability contained growth factors and proteins such as transforming growth factor β1 (TGF‐β1), glial growth factor, and insulin‐like growth factor‐binding protein 4 (IGFBP‐4) that were not detected in the other two lots (Zheng et al., 2006). Moreover, commercially available FBS is only partially characterized. Pedraza et al. showed that FBS from different manufacturers contains calcium oxalate crystals. These crystals can be internalized by murine macrophages and fibroblasts, altering their cell‐cell adhesion and increasing the release of reactive oxygen species (Pedraza et al., 2008). The variations in the quality of serum batches obtained in different seasons can also affect experimental reproducibility by modifying cell growth, viability, and morphology (Barosova et al., 2021).

For these reasons, as well as ethical concerns regarding the origin of FBS (Jochems et al., 2002; Van Der Valk et al., 2004), many researchers have been trying to replace FBS in cell culture media. Among the alternatives to FBS in cell culture are human platelet lysates (Van Der Valk et al., 2010), human AB serum (Heger et al., 2018), bovine ocular fluid (Filipic et al., 2002), fish serum (Rathore et al., 2001), human serum albumin (De Castro et al., 2006), and protein plant extracts (Pazos et al., 2004). These alternatives to FBS overcome the ethical concerns of FBS, however they may be subjected to batch variation as for FBS due to their biological nature, therefore do not completely solve the problems associated with the use of FBS. Human platelet lysates can be a promising alternative to FBS because platelet granules contain an abundance of growth factors and cytokines that support human cell proliferation (Van Der Valk et al., 2010). The use of human platelet lysate started in the 1980s, and it was demonstrated that it can be used for the efficient in vitro culture of fibroblasts (Seidelmann et al., 2021; Umeno et al., 1989), endothelial cells (King & Buchwald, 1984), mesenchymal stem cells (Abdelrazik et al., 2011; Shansky et al., 2019), chondrocytes (Liau et al., 2021), and tumour cell lines (Pons et al., 2019; Wanes et al., 2021). Not only human, but also equine platelet lysate has been used to substitute for FBS in cell culture media and to support the growth of equine MSCs (Chapman et al., 2020; Hagen et al., 2020; Naskou et al., 2019; Russell & Koch, 2016). Another promising alternative to FBS is represented by human serum, which was found to successfully support the proliferation of bone marrow cells (Yamamoto et al., 2003), osteoblasts (Hankey et al., 2001), chondrocytes (Munirah et al., 2008), human Schwann cells (Goodarzi et al., 2014), and both glioma (Clavreul et al., 2009) and melanoma cancer cells (Pandolfino et al., 2010). However, Heger et al. demonstrated that the cell behaviour can be differentially influenced by human serum and FBS. In particular, human serum was able to promote the migration and spheroid formation of the HeLa and SiHa cervical cancer cell lines (Heger et al., 2018). Other studies supported the discrepancies in cell proliferation and differentiation induced by the use of human and bovine serum supplementation (Pisciotta et al., 2012), which could be explained by the differences between the two species (Lau & Chang, 2014). These results underline the importance of standardized culture conditions for preclinical research (Mazlyzam et al., 2008; Pisciotta et al., 2012). Another human blood‐derived product used as to substitute for FBS is human umbilical cord blood plasma (hUCBP), which is rich in soluble growth factors (Bieback et al., 2009; Huang et al., 2011; Pereira et al., 2014). hUCBP was successfully used to support the proliferation, growth, and differentiation of human MSCs (Hong et al., 2014), umbilical cord MSCs, and dental pulp stem cells (Caseiro et al., 2018). Human serum albumin (HSA) has been used as an additional option for FBS in the growth media of two different genetically modified cell lines, murine myoblasts and hamster kidney fibroblasts. HSA at 1% showed the same ability as 10% FBS to support the viability of tested cells; however, when HAS was used the production of erythropoietin (EPO) by myoblasts and the production of brain‐derived neurotrophic factor (BDNF) by fibroblasts was reduced during culture, thus showing that HSA can be used to maintain these cells in culture only for short periods of time (De Castro et al., 2006). One of the main limitations in the use of FBS alternatives is the lack of solid evidence supporting that they do not alter the phenotype of cells, especially in long‐term culture. To address this issue, Fang et al. compared the performances of calf serum‐based alternatives to FBS in the cell culture of head and neck squamous carcinoma cells and dysplastic oral keratinocytes. Calf serum and iron‐supplemented calf serum induced morphological changes, and they were unable to support cell growth in the same way as FBS. On the other hand, Fetalgro, cosmic calf serum, and FetalClone III did not alter cell morphology and promoted cell proliferation more strongly than FBS in head and neck squamous cell lines (Fang et al., 2017). In recent years, chemically defined serum‐free media have been commercialized, i.e. STK2 medium, which contains a lot of essential molecules (epidermal growth factor, basic FGF, glucose, cytokines, vitamins, amino acids, fatty acids, and sodium bicarbonate) and is mostly used for MSC maintenance (Ishikawa et al., 2009). In 2017, Lee et al. demonstrated that STK2 medium was a better option compared to DMEM supplemented with 10% of FBS for the in vitro growth of adipose‐derived stem cells. It was found that the use of STK2 medium held many advantages compared with DMEM supplemented with 10% FBS in terms of adipose‐derived stem cells’ viability and expression of surface markers, such as CD29, CD44, CD73, and CD90, and chondrogenic and osteogenic differentiation (Lee et al., 2017). Although some of these alternatives appear promising, FBS remains the most‐used supplement for cell growth media.

Generally, before being added to the cell medium FBS is heat inactivated at 56°C for 30–60 minutes to destroy complement activity and inactivate potential microbial contaminants (Soltis et al., 1979; Triglia & Linscott, 1980). The removal of complement activity from FBS is required for cell cultures that are sensitive to it, such as immune cells, while it is unnecessary for others (Fante et al., 2021; Pellerin et al., 2021). Heat inactivation does not improve the growth promotion ability of FBS and may have adverse effects; for instance, it can reduce the ability of FBS to promote the cell attachment (Giard, 1987), neurite growth (Bird & Owen, 1998), proliferation, and metabolism of mesenchymal stromal cells (Tonarova et al., 2021). Nevertheless, serum heat inactivation is a routine practice, and it is used in most cell cultures.

## 3 FBS CONTAMINANTS IN CELL‐DERIVED EV SAMPLES AND POSSIBLE SOLUTIONS

As discussed above, the use of FBS has many disadvantages in cell culture. The use of FBS becomes even more crucial regarding studies of EVs because FBS contains FBS‐derived EVs (Théry et al., 2001; Théry et al., 1999), lipoproteins (Busatto et al., 2020), protein aggregates (Mannerström et al., 2019), and nucleic acids (Shelke et al., 2014; Wei et al., 2016) that will be added to the cell culture media and consequently contaminate the cell‐derived EV samples. To overcome/reduce this issue, Théry et al. proposed three possible methods: (i) use serum‐free media; (ii) use media supplemented with only 1% of FBS; and (iii) use media supplemented with FBS previously depleted of EVs (Théry et al., 2006). The use of serum‐free media can be applied only to a few types of cell cultures, such as primary neurons (Roth et al., 2010), epithelial cells (Pazos et al., 2004), and MSCs (Blande et al., 2009), which need a defined media with specific growth factors in order to be properly maintained. The majority of cell lines are usually grown in media supplemented with FBS. To overcome the problem represented by the contamination of FBS‐derived EVs, an option is to grow the cells in medium supplemented with FBS, then exchange the media with serum‐free media, incubate the cells for the conditioning time, and finally collect the conditioned medium. This practice should allow the isolation of pure cell‐derived EVs that are free of exogenous serum‐derived EVs (Bost et al., 2022; Wang et al., 2021). However, this sudden deprivation of serum from the culture medium could cause a shock to the cells, leading to stress responses with consequences for the released EVs in terms of origin, quantity, and content (Witwer et al., 2013). For this reason, it is recommended to monitor these changes and perform all the needed analyses to ensure that the serum deprivation does not affect EV characteristics.

**FIGURE 2:**
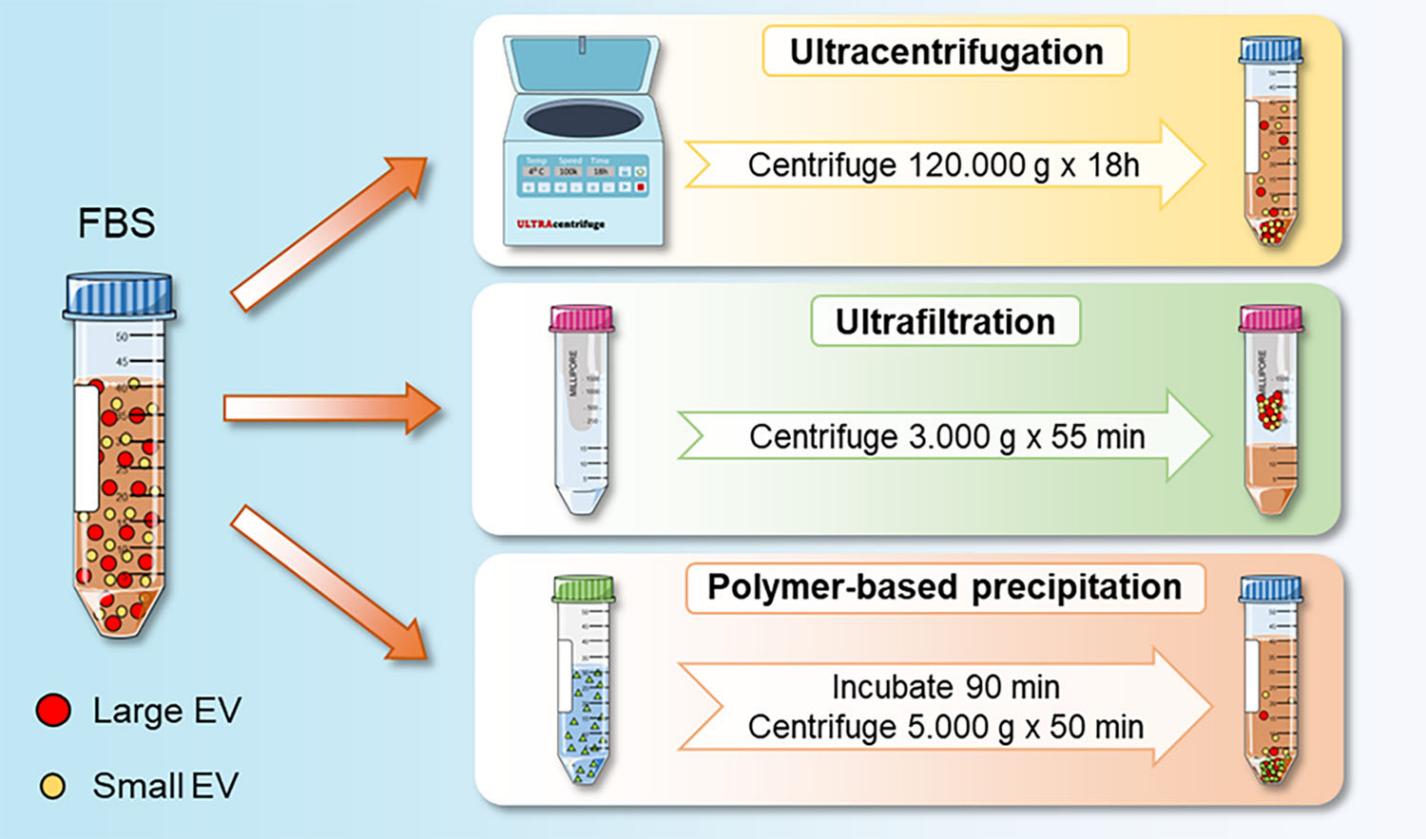
EV‐depletion protocols

Despite the concerns addressed above, as reported by Gardiner et al. (Gardiner et al., 2016) more than 50% of International Society for Extracellular Vesicles (ISEV) respondents use conditioned media supplemented with serum for EV studies, and thus it should be made mandatory to deplete EVs from FBS prior to use for in vitro EV isolation (Théry et al., 2006). A summary of the most‐used protocols to deplete EVs from FBS is shown in Figure 2. Already in the first studies focused on isolating EVs from conditioned media the importance of removing EVs from FBS was highlighted (Raposo et al., 1996; Théry et al., 2001; Théry et al., 1999). One of the first protocols recommended centrifuging the growth media at 110,000 × *g* overnight to eliminate bovine exosomes and protein aggregates (Théry et al., 1999). In the following years, other protocols were suggested using different starting material (FBS or diluted FBS in media) and centrifugation protocols, usually 100,000–120,000 × *g* for 1–18 h (Angelini et al., 2016; Eitan et al., 2015; Ochieng et al., 2009; Shelke et al., 2014; Théry et al., 2006). In 2014, Shelke et al. compared the centrifugation of FBS for a short (1.5 h) and a long period (18 h) to test the efficiency of these two EV depletion protocols. After centrifugation, the supernatant was collected and the EV‐depleted FBS was added to the cell media. To understand the contribution of FBS, EVs were isolated from the media and they found that 1.5 h of centrifugation reduced FBS‐derived EV RNA content by about 50%, while 18 h centrifugation reduced it by 95%. Moreover, the reduction of EV RNA was similar when FBS was diluted before centrifugation. Unfortunately, it is so far not possible to distinguish FBS‐derived EVs from EVs released by cells because both of them show similar characteristics such as round morphology and RNA profile, and thus efficient EV depletion is crucial (Shelke et al., 2014). Shelke et al. demonstrated that 18 h of ultracentrifugation is the best ultracentrifugation time, even if it does not completely eliminate EV contaminants from FBS (Shelke et al., 2014), as was also previously reported by Théry et al. (Théry et al., 2001). This observation was further confirmed some years later comparing EV‐depleted FBS through 18 h ultracentrifugation (UC‐FBS) with commercially available EV‐depleted FBS (Exo‐FBS) (Lehrich et al., 2018). It was found that a significant amount of nano‐sized particles remained in culture media supplemented with both 18 h UC‐FBS and Exo‐FBS, as demonstrated by nanoparticle tracking analysis (NTA). Electron microscopy pictures of culture media supplemented with UC‐FBS showed the presence of some nanoparticles without the typical cup‐shaped morphology of EVs. This indicates that those nanoparticles could likely be lipoprotein particles. Moreover, the FBS contaminants, which were likely represented by both EVs and lipoprotein particles, differed between UC‐FBS, in which particles ranged from 75 to 250 nm, and Exo‐FBS, where the diameter ranged from 75 to 500 nm. The efficiency of EV depletion was about 70% for UC‐FBS and 75% for Exo‐FBS (Lehrich et al., 2018). The UC protocol was also challenged by Pham et al., who further investigated the particles remaining in the FBS after EV depletion through UC and showed that certain nanoparticles are not depleted from FBS after 18 h of ultracentrifugation (Pham et al., 2021).

Because FBS still contains EVs after ultracentrifugation (Lehrich et al., 2018; Pham et al., 2021; Shelke et al., 2014; Théry et al., 2001) other strategies were tested. In 2018, Kornilov et al. proposed an alternative method to depleting EVs from FBS based on ultrafiltration. They used ultra‐15 centrifugal filters and centrifuged FBS for 55 min at 3000 × *g* to obtain ultrafiltration EV‐depleted FBS (UF‐dFBS), which was compared with EV‐depleted FBS through ultracentrifugation (UC‐dFBS) and commercially available Exo‐FBS. Following the EV‐depletion protocols, they isolated and characterized the remaining EVs in all tested samples. Transmission electron microscopy showed that UF‐dFBS did not contain any remaining EVs compared to SBI‐dFBS and UC‐dFBS, and they could not detect Hsp70 or CD71 using western‐blot in UF‐dFBS, while these two EV markers were present in SBI‐dFBS and UC‐dFBS. Finally, to ensure that the ultrafiltration protocol did not affect the capability of FBS to maintain cell growth or modify the cell phenotype, they supplemented the media with different types of FBS (UF‐dFBS, UC‐dFBS, SBI‐dFBS, and unprocessed FBS). They demonstrated that adipose‐derived MSC proliferation and expression of surface markers, such as CD73, CD90, and CD105, were similar in all of the tested conditions. They also analysed the proliferation of three cancer cell lines – HSC3, PC‐3, and HOS143b – and found that media supplemented with all types of tested FBS had a similar capability to support cell growth (Kornilov et al., 2018). Another group confirmed the efficiency of the ultrafiltration protocol for EV depletion and compared it with the other two protocols – sonication and polymer‐based precipitation. Among these methods, polymer precipitation, which was performed by incubating FBS with PEG 4000 for 90 min and then centrifuging at 5000 × *g* for 50 min, was the best solution with more than 91% EV depletion versus 85% for ultrafiltration and 70% for sonication. Moreover, the EV depletion through polymer precipitation did not affect the morphology, proliferation, or immunophenotype of adipose‐derived MSCs (Haghighitalab et al., 2020).

Finally, another alternative emerging in recent years is represented by commercially available EV‐depleted FBS. Nevertheless, the protocols used to deplete FBS from EVs are not described in detail, and the purity of these products is usually evaluated through limited techniques, such as NTA and western‐blot, and never showing electron microscopy images as proof of EV depletion. Because some studies have demonstrated that commercial EV‐free FBS can contain contaminants (Kornilov et al., 2018; Lehrich et al., 2018), such materials should be used with caution for in vitro EV studies.

A point that should be clarified in the protocols of FBS preparation for in vitro EV studies regards the heat‐inactivation procedure. Many studies do not specify whether FBS was heat inactivated (usually 56°C for 30–60 min under agitation (Bruinink et al., 2004; Giard, 1987; Leshem et al., 1999; Pellerin et al., 2021; Soltis et al., 1979)) prior to use, and when specified the authors usually do not explain if the heat inactivation procedure was performed before or after the EV depletion or how the heat inactivation was performed in terms of temperature and time. To better standardize EV research, information concerning the use of FBS in cell cultures should be added in the methods section of published works.

Bovine EVs are not the only contaminants in cell‐derived EVs, as shown by Shelke et al., and FBS also contains a huge amount of RNA (Shelke et al., 2014). In another study, Wei et al. further investigated FBS and showed the presence of both coding and non‐coding RNA that persisted after the EV depletion and thereby could be co‐isolated with cell‐derived EVs (Wei et al., 2016). In particular they showed that some miRNAs, such as miR‐122 and miR‐451a, were enriched in EVs released by glioma cells. The investigated miRNAs were not expressed in those cells, thus suggesting that their presence could be due to exogenous contaminants. To prove this, the same cell line was cultured in serum‐free media and the analysis of the resulting EVs revealed that miR‐451a was not detected. These observations have questioned previous studies focusing on EVs and RNA because bovine RNA contaminants could lead to false results of RNA enrichment in cell‐derived EVs. After 24 h of ultracentrifugation, the authors showed that the amount of RNA was reduced by 20–30% and that the total remaining RNA (not only enclosed in EVs but also associated with proteins) could co‐precipitate with EVs during isolation from both human and mouse cell culture media. Moreover, because the RNA maps to human and mouse genomes, it cannot be distinguished from human or mouse RNA, thereby confounding studies of EV RNA profiles (Wei et al., 2016). In 2018, Driedonks et al. proposed a way to optimize EV‐depletion protocols to reduce the interference of FBS‐derived RNA in EV samples in vitro (Driedonks et al., 2019). They demonstrated that serum‐derived RNA precipitated more efficiently when FBS was previously diluted in cell culture media compared to un‐diluted FBS. By comparing the amount of some specific miRNAs and cytoplasmic and nuclear RNAs in EV‐depleted supernatant and non‐depleted medium, they found that the efficiency of RNA depletion could vary depending on the RNA types. In particular, several members of Y‐RNA family, 7SL, and miR‐142 were efficiently depleted from FBS after ultracentrifugation; while miR‐1246, miR‐148a, miR‐122 and miR‐423 were not depleted at all, probably due to their association with lipoprotein particles or ribonucleoproteins. Although not all RNAs can be efficiently depleted from FBS using ultracentrifugation, they demonstrated that using differential ultracentrifugation followed by density separation, the amount of contaminant RNA in cell‐derived EVs could be reduced. According to these findings, unconditioned medium should be used as control in the studies focused on EV RNA content with the aim to determine the background RNA levels to be subtracted to the RNA found in EV samples (Driedonks et al., 2019). A possible strategy to overcome this bovine RNA contamination could be the use of serum‐free media supplemented with chemically defined constituents. Nevertheless, the presence of exogenous miRNAs in cell‐derived EVs was also reported in cells maintained in serum‐free media (Auber et al., 2019). It has been demonstrated that EVs released by primary mouse oligodendrocytes harvested in serum‐free media were enriched in miR‐122 and miR‐451a (expressed by liver and blood cells respectively (Landgraf et al., 2007; Ludwig et al., 2016)) and miR‐1246, which is not produced by rodent cells (Zhang et al., 2011). Analysing the pellet resulting from differential centrifugation of unconditioned serum‐free media revealed that miRNA contamination came from catalase, which is found in the NS21 supplement (Auber et al., 2019) as well as in FBS. Because it was not possible to completely eliminate exogenous miRNAs by using RNase A and Triton‐X100 (Auber et al., 2019), as suggested by Driedonks et al. for bovine RNA contaminants (Driedonks et al., 2019), it is strongly recommended to perform an analysis of the background miRNAs (isolated from the unconditioned media) in studies of EV miRNA profiling. In addition to EVs, it has been shown that commercial serum‐free media is full of protein aggregates that can “contaminate” the cell‐derived EV pellet (Witwer et al., 2013).

## 4 THE UNSUSPECTED FUNCTIONS OF FBS‐DERIVED EVS IN CELL CULTURES

Although initially described as ‘garbage bags’, EVs are now widely studied by many research groups because they play key roles in cell‐cell communication (Tkach & Théry, 2016). They act as vehicles for biological information, and they can transfer this information to recipient cells and modify their phenotypes (H Rashed et al., 2017). The EVs’ effects on recipient cells depend on the EV content, which differs according to the EV originating cell type. Consequently, FBS‐derived EVs also contain specific molecules that can determine biological activities. Also, although the physiological functions of FBS‐EVs have not been investigated yet, many studies have focused on the mechanism underlying the role of EVs in bovine embryo development, such as the effect on blastocyst rate and cell apoptosis (Asaadi et al., 2021; Dissanayake et al., 2020; Pavani et al., 2018, 2020), because it is known that EVs mediate embryo‐maternal crosstalk (Bridi et al., 2020). Pavani et al. isolated EVs from conditioned media of bovine embryos and demonstrated that EVs can be internalized by embryonic cells by crossing the zona pellucida. They found that bovine embryo EVs were functionally active because they were able to promote the development of embryos, increase their quality, and reduce mortality (Pavani et al., 2018). Given the potential of bovine embryo‐derived EVs in supporting embryo development and their ability to mediate this embryo‐maternal crosstalk, it is not surprising that EVs derived from FBS may also affect the biological properties of mammalian cells in culture. The interaction between FBS‐derived EVs and cultured cells is supported by several studies (Eitan et al., 2015; Ochieng et al., 2009). It was demonstrated that FBS‐EVs can be successfully taken up by human breast carcinoma cells (Ochieng et al., 2009), murine neuroblastoma cells, human glioblastoma cells, and human kidney cells (Eitan et al., 2015). The effects of EVs on the growth, phenotype, and EV release by recipient cells will be discussed in the next sections.and are listed in Table 1.

**TABLE 1:** The effect of FBS‐derived EVs on cell cultures

Cell growth			
Target cells		Functional effects	Reference
Human	Breast carcinoma	Promote anchorage‐independent growth through the ERK signalling pathway	(Ochieng et al., 2009)
	Glioblastoma	Enhance the growth rate and viability of cells	(Eitan et al., 2015)
	Kidney		
	Cervical carcinoma		
	Neuroblastoma		
	Skeletal muscle	Stimulate cell proliferation	(Aswad et al., 2016)
	Cardiac progenitor	Promote cell proliferation by increasing the gene expression of Ki‐67, Talin‐1, and Vinculin	(Angelini et al., 2016)
Mouse	Neuroblastoma	Enhance the growth rate and viability of cells	(Eitan et al., 2015)
	Skeletal muscle	Stimulate cell proliferation by up‐regulating CCND1 and SIRT1 gene expression	(Aswad et al., 2016)
	Breast cancer cells	Increase cell proliferation	(Gstraunthaler, 2003)
	Macrophages		
	Astrocytes	Enhance cell proliferation and viability	(Lehrich et al., 2018)
Cell phenotype			
Target cells	Functional effects	Reference	
Human	Airway epithelial cancer	Promote cell migration	(Shelke et al., 2014)
	T lymphocytes	Reduce HIV infectivity	(Liao et al., 2017)
	Skeletal muscle	Impair cell differentiation	(Aswad et al., 2016)
	Cardiospheres	Increase the yield and the size of cardiospheres	(Angelini et al., 2016)
Mouse	Peritoneal macrophages	Decrease pro‐inflammatory cytokine levels	(Beninson & Fleshner, 2015)
	Skeletal muscle	Impair cell differentiation	(Aswad et al., 2016)
	Breast cancer	Increase the resistance to enoxacin and bis‐enoxacin	(Vracar et al., 2018)
	Astrocytes	Preserve proper cell morphology	(Lehrich et al., 2018)
EV release			
Target cells	Functional effects	Reference	
Human	Breast cancer	Enhance EV release through recycling	(Ochieng et al., 2009)
Mouse	Skeletal muscle	Promote EV formation and vesicular trafficking	(Aswad et al., 2016)

### 4.1 FBS‐derived EVs promote cell growth in vitro

EVs present in FBS are similar to EVs released by cells (Aswad et al., 2016; Mannerström et al., 2019), and thus, as for cell‐derived EVs, they may contain proteins and factors responsible for cell growth‐promoting activity. This makes bovine EVs a possible vehicle to transport these bioactive molecules to cultured cells. A study in favour of this hypothesis showed that FBS‐derived EVs carry fetuin‐A (Ochieng et al., 2009), a serum protein that positively regulates the growth of lung cancer cells (Kundranda et al., 2005) and mediates EV biogenesis (Watson et al., 2012). It was found that FBS‐derived EVs could be taken up by breast carcinoma cells and that they were able to promote anchorage‐independent growth more efficiently when compared to exosome‐free medium. The growth‐promoting ability of FBS‐derived EVs was mediated through the ERK signalling pathway (Ochieng et al., 2009). In line with this finding, another study demonstrated that FBS‐EVs could support the growth of human and mouse cell lines (Eitan et al., 2015). The growth rates of four human cell lines (glioblastoma, kidney, cervical carcinoma, and neuroblastoma) and one murine neuroblastoma cell line in media supplemented with EV‐depleted FBS were compared to media supplemented with unprocessed FBS. It was demonstrated that the growth rates and viability of human kidney, cervical carcinoma, neuroblastoma cells, and mouse neuroblastoma cells were strongly reduced in EV‐depleted media compared to media supplemented with unprocessed FBS. The addition of bovine EVs to EV‐depleted media rescued the growth reduction, thus confirming the involvement of FBS‐EVs in supporting cell growth. Furthermore, the EV depletion also negatively affected the viability of human glioblastoma and kidney cells and mouse neuroblastoma cells when human serum, a promising alternative to FBS (Heger et al., 2018), was used (Eitan et al., 2015). Moreover, mouse neuroblastoma cells cultured in media supplemented with EV‐depleted human serum showed higher sensitivity to tunicamycin, an activator of the unfolded protein response. The accumulation of unfolded protein in the endoplasmic reticulum (ER) causes ER stress and activates the unfolded protein response, which can lead to cell death (Hetz et al., 2013). These results underline that serum‐derived EVs may protect the cells from ER stress by decreasing the sensitivity to tunicamycin (Eitan et al., 2015). It was found that bovine EVs can be internalized by human glioblastoma and kidney cells and murine neuroblastoma cells and end up in the lysosomal compartment. Because it is known that lysosomes are subcellular organelles involved in the digestion of macromolecules, bovine EVs may be internalized in the lysosomes to provide amino acids, lipids, and other biosynthetic precursors to the cell (Eitan et al., 2015).

Previous works showed that the geographic origin of FBS (where in the world the FBS comes from) can influence the proliferation of both muscle cells (Khodabukus & Baar, 2014) and cardiac progenitor cells (CPCs) (Chimenti et al., 2014) differently. In particular, FBS‐derived EVs were shown to stimulate the proliferation of both mouse and rat muscle cell lines, as well as human primary myoblasts (Aswad et al., 2016). After a culture period of 3 days in media supplemented with unprocessed FBS and UC‐FBS, the proliferation of muscle cells decreased in the absence of serum‐derived EVs as well as the gene expression of Cyclin D1 and Sirt1, both of which play important roles in cell proliferation (Rathbone et al., 2009; Sherr, 1995). It was also found that the cell proliferation of CPCs was negatively affected by EV‐depleted media (both UC‐FBS and commercially available Exo‐FBS) compared to EV‐containing media, while the reintroduction of FBS‐EVs rescued cell growth in a dose‐dependent manner. The reduction in the cell proliferation rate could be explained by the downregulation of genes involved in this process, such as *Ki‐67*, *Talin‐1*, and *Vinculin*, as observed in cells grown in EV‐depleted media compared to cells cultured in media supplemented with undepleted FBS (Angelini et al., 2016).

Murine breast cancer cells (4T1) and murine macrophages (RAW264.7) are two cell lines that require the presence of FBS in order to be maintained in culture (Christy et al., 2020). However, it was found that 4T1 and RAW264.7 cells could grow in serum‐free media supplemented with FBS‐EVs. In fact, while these two cell lines were not able to proliferate in serum‐free media, their viability and cell proliferation increased when FBS‐derived EVs were added to serum‐free media (Christy et al., 2020). Moreover, Lehrich et al. studied the effects of FBS‐derived EVs on cell growth and viability of primary astrocytes (Lehrich et al., 2018) and found that the proliferation rate of astrocytes decreased when media was supplemented with UC‐FBS or commercially available EV‐free FBS. In addition, EV‐depleted media negatively affected the cell viability of primary astrocytes because the count of viable cells was lower in EV‐depleted media compared to EV‐containing media (Lehrich et al., 2018). Taken together, these results showed how EV‐derived FBS can influence cell growth, thus underlining the importance of these particles in EV studies based on cell lines. However, the EV depletion process through UC protocol could lead to a partial loss of some growth factors and other FBS components that could end up with the EV pellet; for this reason, the effects of EV depleted FBS may be ascribed not only to the elimination of FBS‐derived EVs but also to the possible reduction of other constituents caused by the ultracentrifugation.

### 4.2 FBS‐derived EVs affect the cell phenotype

One of the first studies demonstrating that EVs present in FBS could affect the phenotype of cells in culture came in 2014 with the work of Shelke et al. (Shelke et al., 2014). The authors demonstrated that EVs from FBS could be internalized by airway epithelial cancer cells and promote their migratory ability in a dose‐dependent manner, supporting the idea that FBS‐EVs can affect cell behaviour (Shelke et al., 2014). Because EVs isolated from the human placenta are involved in foetal protection by exerting immunosuppressing functions (Bai et al., 2022; Stenqvist et al., 2013), FBS‐derived EVs might possess similar activities and mediate cross‐species protection of cell cultures. In a work by Beninson and Fleshner, the immunomodulatory properties of FBS‐EVs on cell cultures were evaluated (Beninson & Fleshner, 2015). The authors compared the levels of pro‐inflammatory cytokines, such as interleukin 1β (IL‐1β), interleukin 6 (IL‐6), and tumour necrosis factor α (TNF‐α), released by primary murine peritoneal macrophages treated with LPS and cultured with 10% FBS or EV‐depleted FBS (Ciesielska et al., 2021). They found that the release of IL‐1β was decreased when macrophages were cultured in media with undepleted FBS compared to media with EV‐depleted FBS, and the re‐introduction of bovine EVs in EV‐depleted media reduced IL‐1β levels in a dose‐dependent manner. Moreover, the addition of EV‐derived FBS downregulated IL‐6 and TNF‐α levels in a dose‐dependent manner (Beninson & Fleshner, 2015). The correlation between FBS‐EVs and immune response was further supported by another group that highlighted the role of bovine serum EVs in HIV infectivity in vitro (Liao et al., 2017). Two T‐lymphocytic cell lines, H9 and PM1, were grown for 7 days in media supplemented with unprocessed FBS, UC‐FBS, or commercial EV‐depleted FBS and then infected with HIV‐1. The release of HIV in EV‐depleted media was higher than in EV‐derived FBS‐containing media for both cell lines; however, the re‐introduction of EV‐derived FBS reduced the release of HIV. This could be due to the upregulation of genes involved in lipid synthesis pathways in T‐lymphocytes grown in EV‐depleted media, which may lead to an increase in EV release and virion biogenesis, thus demonstrating that bovine EVs affected HIV production and infectivity (Liao et al., 2017).

It was also found that FBS‐derived EVs could impair the differentiation of skeletal muscle cells (Aswad et al., 2016). In particular, it was shown that myoblasts grown in medium containing EV‐depleted FBS had lower levels of myogenin, a protein involved in myotube formation, and produced fewer myotubes than cells cultured in EV‐derived FBS‐containing media. Moreover, cells maintained in media supplemented with EV‐depleted FBS had higher levels of myostatin, an inhibitor of muscle cell differentiation (Aswad et al., 2016), than cells grown in media containing unprocessed FBS, thus confirming the involvement of bovine FBS in skeletal muscle cells differentiation (Aswad et al., 2016). Bovine serum EVs also gave a contribution to human cardiosphere culture, as demonstrated in the work by Angelini et al. (Angelini et al., 2016). The authors found that the formation of cardiospheres was negatively influenced by the absence of bovine EVs in cell culture media in terms of yield and cell size. The gene expression analysis revealed that the effects of EV‐derived FBS could be explained by their ability to modulate the levels of genes involved in extracellular matrix production (Angelini et al., 2016). Interestingly, the presence of FBS‐EVs in cell growth media was also correlated with the resistance of murine breast cancer cells to enoxacin and bis‐enoxacin (Vracar et al., 2018), two possible therapeutic agents against cancer and bone diseases (Liu et al., 2017; Melo et al., 2011). Finally, Lehrich et al. found that primary astrocytes grown in culture media supplemented with UC‐FBS and commercial EV‐depleted FBS showed size reductions compared to cells grown in media supplemented with unprocessed FBS, and they more easily detached from the flask (Lehrich et al., 2018).

### 4.3 FBS‐derived EVs can influence the release of EVs

The release of EVs by cells in vitro can be influenced by several factors such as culture media composition and the use of particular supplements (Théry et al., 2018). Moreover, some parameters of the protocol used for EV isolation, such as the centrifugation time and the rotor type, may also impact the yield and purity of isolated EVs (Théry et al., 2018). Interestingly, the research group of Andaloussi found that human and mouse neuroblastoma cells released a larger amount of EVs in serum‐free media compared to media supplemented with EV‐depleted FBS (Li et al., 2015). Moreover, EVs isolated from murine cells cultured in serum‐free media showed a different protein cargo compared to EVs released in EV‐depleted FBS media. Among the proteins differentially enriched in EVs in serum‐free conditions there were small GTPases, such as Rab27a and Rab27b, which are involved in MVB fusion with the plasma membrane (Pfeffer, 2010), and ARF6, which takes part in EV biogenesis (Ghossoub et al., 2014). The increased expression of these proteins could explain the enhancement of the EV release by cells maintained in serum‐free media (Li et al., 2015). Recently, the same group evaluated EV production in different growth conditions by using HEK293T cells that were transduced to stably express a tetraspanin‐TLuc fusion protein (Bost et al., 2022). They found that HEK293T cells released a larger amount of EVs in Opti‐MEM than in DMEM supplemented with 10% FBS, and they demonstrated that albumin and globulin, two well‐represented proteins contained in FBS, were responsible for the reduced EV production. This difference in EV release could be explained by the upregulation of genes involved in EV production pathways, such as biosynthetic processes of sphingolipid and ceramide in cells grown in Opti‐MEM (Bost et al., 2022).

The use of FBS or other supplements of animal origin limits the translation of EVs studies to clinical application (Heiskanen et al., 2007; Sundin et al., 2007; Tuschong et al., 2002), and for this reason many commercial xeno‐free media have been developed (Chase et al., 2012; Oikonomopoulos et al., 2015; Patrikoski et al., 2013), although these might influence the properties of cultured cells and their derived EVs. Bobis‐Wozowicz et al. demonstrated that different xeno‐free media affected the phenotype of human umbilical cord MSCs, thus impacting their functional properties (Bobis‐Wozowicz et al., 2017). They found that when the media was supplemented with the MSCGM‐CD™ Bullet Kit, the MSC proliferation, metabolic activity, and surface antigen profile were optimal, which was different from MSC behaviour in media with StemPro^®^ MSC SFM Xenofree in which they differentiated into osteocytes, chondrocytes, and adipocytes. These changes in cellular behaviour affected the cargo and consequently the biological properties of the released EVs. The authors demonstrated that EVs isolated from cells maintained in media supplemented with StemPro^®^ MSC SFM Xenofree or MSC NutriStem^®^ XF Basal Medium + MSC NutriStem^®^ XF Supplement Mix were enriched in cardiac regulatory and pro‐angiogenic miRNAs, thereby favouring cardiomyogenesis and angiogenesis. Nevertheless, the immunomodulatory functions of MSC‐derived EVs were preserved in media plus StemPro^®^ MSC SFM Xenofree because these media inhibited the proliferation of human peripheral blood mononuclear cells (Bobis‐Wozowicz et al., 2017).

As discussed in the previous section, the heat inactivation of FBS is a practice widely used in cell culture laboratories (Soltis et al., 1979; Triglia & Linscott, 1980). However, in EV studies there is a need to standardize this procedure because it could affect the quality and purity of cell‐derived EV samples, as demonstrated in a recently published study (Frigerio et al., 2021). In this work, EVs were isolated from COVID‐19 patient serum to investigate the impact of heat inactivation on EV isolation. The serum was heat‐treated at 56°C for 30 min with the aim to inactivate SARS‐CoV2, and then EVs were isolated. EVs isolated from heat‐inactivated serum were compared to untreated serum, and it was found that the number of particles isolated from heat‐inactivated serum was higher compared to untreated serum, but so was the protein concentration, suggesting an increase in contaminants co‐isolated with EVs. This evidence underlined that heat inactivation could affect the composition of serum, increasing the amount of contaminant molecules/particles in the EV samples (Frigerio et al., 2021).

**FIGURE 3:**
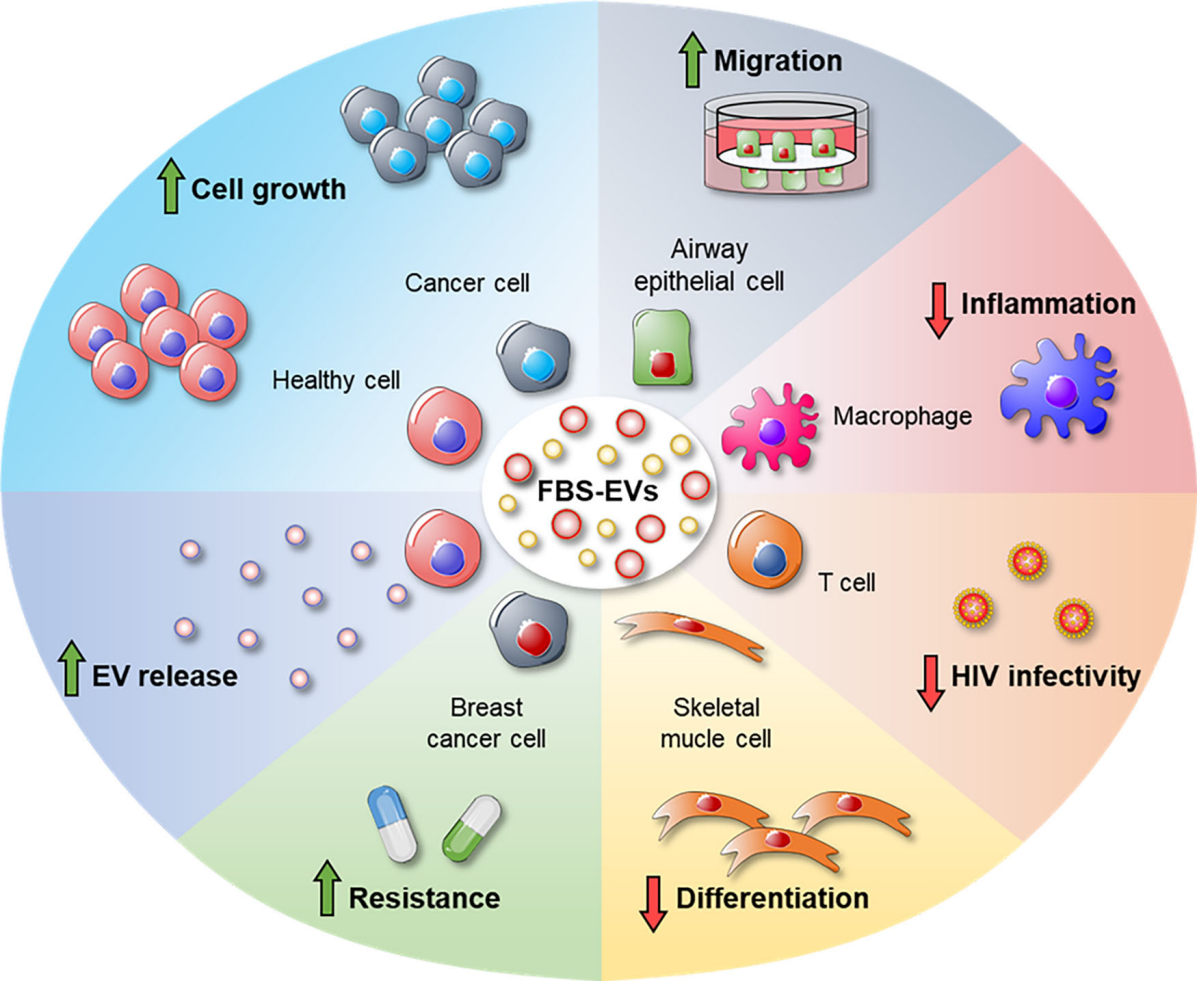
The biological functions of FBS‐EVs on cell cultures

Given the effects of EV depletion from FBS on cell growth and phenotype, the use of EV‐depleted FBS in cell culture media might also have an influence on the release of EVs by cultured cells. Although the impact of FBS‐derived EVs on EV secretion in vitro has been poorly studied, probably due to the difficulties in differentiating cell‐derived EVs from FBS‐EVs (Aswad et al., 2016; Mannerström et al., 2019), some studies have highlighted the possible effects of bovine EVs in this process (Aswad et al., 2016; Ochieng et al., 2009). Ochieng et al. demonstrated that bovine EVs, once up taken by breast cancer cells, were re‐cycled through the endosomal compartment by exiting from a donor cell and being internalized by another cell. This re‐cycling mechanism results in the secretion of EVs with a unique cargo that derives partially from EV‐derived FBS. These results showed that EV‐derived FBS favours EV release (Ochieng et al., 2009). FBS‐EVs can also influence EV formation and vesicular trafficking, as demonstrated by Aswad et al. (Aswad et al., 2016). The authors found that the expression of *VPS37B* and *VPS4A*, two genes involved in multivesicular body formation (Huttunen et al., 2021; Okumura et al., 2013), in myoblasts cultured in EV‐depleted medium was lower than in EV‐containing medium, thus demonstrating that bovine EVs may promote cellular EV production in vitro (Aswad et al., 2016). Overall, the effects of FBS‐derived EVs on cell cultures are schematized in Figure 3.

## 5 CONCLUSIONS

As the field of EVs is continuously expanding, both in clinical and pre‐clinical applications, there is a growing need to standardize the way in which cells are cultured. A major limitation of EV studies in vitro is the use of FBS. FBS is an essential supplement for cell culture media, but it contains EVs that contaminate cell‐derived EVs. The most‐used protocol for FBS EV depletion is ultracentrifugation, but even though it has been optimized over the years it does not seem to completely eliminate exogenous EVs and macromolecules. Although many works underlined the persistence of FBS EVs in the culture media after EV depletion, a direct comparison between complete FBS and EV depleted FBS according to the MISEV guidelines is still missing. It could be helpful for the EV community to better characterize these two samples using all the techniques recommended for EV characterization to obtain a comprehensive overview of the differences between unprocessed and EV depleted FBS. Moreover, during the EV depletion process some non‐vesicular particles of FBS may potentially end up with the EVs, thereby UC could lead to the loss of other FBS components which could explain some side effects showed by the use of UC‐FBS. New and promising EV‐depletion methods are emerging, such as ultrafiltration and polymer‐based precipitation, which may represent viable solutions to this problem. Given that EV depleted FBS could affect cell growth (Eitan et al., 2015) and phenotype (Aswad et al., 2016), we recommend taking it into account when cells are cultured in EV depleted media. Since EV release and content can vary depending on the cell state (O'Brien et al., 2020), it is essential to ensure that cell maintained in EV depleted media are not negatively influenced by EV depletion. Moreover, as highlighted from many works (Driedonks et al., 2019; Shelke et al., 2014; Wei et al., 2016), the analyses of cell‐derived EV content can be distorted by the co‐isolation of FBS contaminants with the EV samples. For this reason, it is strongly suggested to evaluate the background of the analytes of interest, such as RNAs, by analysing unconditioned media supplemented with FBS. Following these recommendations may help to overcome the FBS limitations in EV studies, thereby increasing the validity of the in vitro results and their translation to in vivo applications.

## AUTHOR CONTRIBUTIONS

O.U. and R.C. wrote the manuscript. ROB and RC discussed and edited the manuscript. All authors approved the final manuscript. Ornella Urzì: Conceptualization;  Writing – original draft. Roger Olofsson Bagge: Funding acquisition; Project administration; Writing – review & editing. Rossella Crescitelli: Conceptualization; Investigation; Methodology; Supervision; Writing – original draft; Writing – review & editing.

## CONFLICTS OF INTEREST

R.C. has developed multiple EV‐associated patents for putative clinical utilization: US20200088734A1, United States; WO2020146390A1, WIPO (PCT). R.C. owns equity in Exocure Bioscience Inc. ROB has received institutional research grants from Bristol‐Myers Squibb (BMS) and SkyLineDx, speaker honorarium from Roche and Pfizer and has served on advisory boards for Amgen, BD/BARD, Bristol‐Myers Squibb (BMS), Merck Sharp & Dohme (MSD), Novartis, Roche and Sanofi Genzyme.
