# Correction to “The dark side of foetal bovine serum in extracellular vesicle studies”

**DOI:** 10.1002/jev2.12424

**Published:** 2024-03-15

1

Urzì, O., Olofsson Bagge, R., & Crescitelli, R. (2022). The dark side of foetal bovine serum in extracellular vesicle studies. *Journal of Extracellular Vesicles*, 11, e12271. https://doi.org/10.1002/jev2.12271

In the originally published article, the year in the received/revised/accepted dates was incorrectly given as 1912. This has been corrected in the online version of the article.

The citation has also been updated to include the “e” in the elocation ID, which was inadvertently left out. This has been corrected in the online version of the article.

We have also corrected the tagging of author Roger Olofsson Bagge to correctly reflect their forename and surname.

We apologize for these errors.

